# Understanding the molecular mechanism for the differential inhibitory activities of compounds against MTH1

**DOI:** 10.1038/srep40557

**Published:** 2017-01-11

**Authors:** Mian Wang, Shuilian Zhou, Qing Chen, Lisheng Wang, Zhiqun Liang, Jianyi Wang

**Affiliations:** 1 State Key Laboratory for Conservation and Utilization of Subtropical Agro-Bioresources, Guangxi University, Nanning 530004, People’s Republic of China; 2 College of Life Science and Technology, Guangxi University, Nanning 530004, People’s Republic of China; 3 School of Chemistry and Chemical Engineering, Guangxi University, Nanning 530004, People’s Republic of China

## Abstract

MTH1 can hydrolyze oxidized nucleotides and is required for cancer survival. The IC_50_ values were 0.8 nM for TH287 with a methyl substitution, 5.0 nM for TH588 with a cyclopropyl substitution, and 2.1 μM for TH650 with an oxetanyl substitution. Thus, it is very significant to understand inhibitory mechanisms of these structurally similar compounds against MTH1 and influences of the substituent on the bioactivities. Our MD researches indicate that TH287 maintains significant hydrogen bonds with Asn33 and Asp119, stabilizes the binding site, and induces MTH1 adopt a closed motion, leading to a high inhibitory activity. When bound with TH588, the binding site can be partially stabilized and take a semi-closed state, which is because the cyclopropyl group in TH588 has larger steric hindrance than a methyl group in TH287. So TH588 has a slightly reduced inhibitory activity compared to TH287. TH650 induces greater conformation fluctuations than TH588 and the binding site adopts an opening state, which is caused by the large bulk of oxetanyl group and the interference of solvent on the oxetanyl substituent, leading to the lowest inhibitory activity. Thus, the inhibitory activity follows a TH287 > TH588 > TH650 trend, which well matches with the experimental finding.

The MutT homolog 1 (MTH1) can efficiently hydrolyze oxidized nucleoside triphosphates to nucleoside monophosphates^1^,^2^,^3^,^4^,^5^. For example, MTH1 can hydrolyze 8-oxo-rATP, 8-oxo-dATP, 8-oxo-rGTP, 8-oxo-dGTP, 2-OH-dATP and 2-OH-rATP^6^,^7^,^8^,^9^,^10^,^11^. Therefore, MTH1 can prevent the incorporation of these oxidized nucleoside triphosphates into DNA, and thus effectively reduce the mutation and cell death. It is reported that MTH1 is not necessary for the normal cells but significant for the survival of the cancer cells, because cancer cells have a high level of reactive oxygen species which can cause the oxidization of nucleoside triphosphates^12^. Therefore, MTH1 is a promising target of cancer treatment, and the inhibition of MTH1 by small molecules can suppress the tumor.

Many experimental and theoretical efforts were devoted to investigate the substrates binding to MTH1. The crystal structures of human MTH1 bound with 8-oxo-dGMP (pdb code: 3ZR0) clearly shows that the base of dGMP recognizes MTH1 via H-bonding to Asn33, Asp119 and Asp120^7^. This binding mode provides some cues that the base analogues may be potential lead compounds to competitively inhibit MTH1. The half maximal inhibitory concentration (IC_50_) means the concentration of the inhibitor which is required to inhibit a biological or biochemical function, and thus IC_50_ is usually used to measure the effectiveness in inhibiting biological or biochemical functions^13^. The crystal structures of chiral crizotinib binding to MTH1 (pdb codes: 4C9X for (S)-crizotinib and 4C9W for (R)-crizotinib) have been reported^14^, and (S)-crizotinib is a potent inhibitor of MTH1 (IC_50_ 72 nM) whereas (R)-crizotinib is almost inactive (IC_50_ 1375 nM). Some groups further unravel the chirality-selectivity inhibition mechanism of MTH1 using molecular dynamic simulations. Sun and colleagues revealed that Asn33 and Trp117 touched or inserted into the van der Waals surface of (R)-crizotinib which contributed to the unfavorable binding of (R)-crizotinib with MTH1 protein^15^. Niu and co-workers illuminated that Tyr7, Phe27, Phe72 and Trp117 were significant for the selective inhibition of (S)-crizotinib on MTH1^16^. Zhou and co-workers demonstrated that (S)-crizotinib could induce larger conformation fluctuation on MTH1 than (R)-crizotinib^17^.

Recently, a potent MTH1 inhibitor TH287 has been reported with low IC_50_ value of 0.8 nM^18^ (shown in Fig. 1). When the methyl group in TH287 is replaced by the cyclopropyl moiety in TH588, the inhibitory activity is slightly reduced (IC_50_ 5.0 nM)^18^. Surprisingly, the replacement of the methyl group by an oxetanyl substituent in TH650 greatly reduces the inhibitory activity (IC_50_ 2.1 μM)^18^. The cell biology assays show that TH287 and TH588 selectively and effectively kill cancer cell lines, whereas TH650 is not toxic to any cells tested^18^. In the crystal structures of MTH1 bound with TH287 (PDB code 4N1T) and TH588 (PDB code 4N1U)^18^, TH287 and TH588 H-bond to Asn33, Asp119 and Asp120 in the active site of MTH1. However, the dynamic interactions between these three analogues and MTH1 as well as the effects of substituent on the inhibitory activities are still hiding in the shadow. To address why the inhibitory activity follows a TH287 > TH588 > TH650 trend, four models (the *apo* MTH1, the TH287-MTH1 complex, the TH588-MTH1 complex and the TH650-MTH1 complex) are comparatively investigated by molecular dynamics simulations. Our work could provide new insights into the inhibition mechanism of these three analogues against MTH1 and the influences of substituent on the inhibitory activities, and supply some information to design novel therapeutic agents against MTH1 protein.

## Methods

The crystal structures of the MTH1 bounded with two inhibitors TH287 and TH588 were obtained from the RCSB protein data bank: 4N1T for TH287-MTH1 complex, 4N1U for TH588-MTH1 complex^18^. The TH588-MTH1 complex was crystallized in a dimer form, in which chain A contained residues 3–155 while chain B included residues 2–155. The residue number of chain B in the TH588-MTH1 complex is consistent with the counterpart in the TH287-MTH1 complex. Besides, chain B of the TH588-MTH1 complex can align well with the TH287-MTH1 complex (Cα RMSD 0.3 nm). Therefore, chain B was selected as the initial structure of the TH588-MTH1 complex. The initial structure of *apo* MTH1 is generated by deleting the ligand from the TH287-MTH1 complex. The structure of TH650 is created via replacing the cyclopropyl group of TH588 by an oxetanyl group using GaussianView 5.0 software. As the bulk volume of TH650 is similar to TH588, the initial structure of TH650-MTH1 complex is generated through replacing TH588 by TH650 in the crystal structure of the TH588-MTH1 complex. All crystal water molecules were kept in the initial structures. Molecular dynamics (MD) simulations were carried out with the GROMACS 4.5.5 package^19^,^20^,^21^. The AMBER99SB-ILDN force field was applied^22^. The topology files of the three ligands were generated by antechamber and ACPYPE^23^,^24^. The atomic partial charges of ligands were obtained through B3LYP/6–31 G(d, p) single-point calculation with the restrained electrostatic potential (RESP)^25^ method using GAUSSIAN09 program^26^. Each protein or complex was solvated with TIP3P water molecules^27^,^28^, and placed in a rectangular box with the minimal distance of 1.2 nm to the wall. In order to maintain an electroneutral system and mimic the physiological environment, counterions were added to systems: 38 Na^+^ and 31 Cl^−^ ions for the *apo* MTH1 or the TH287-MTH1 complex, and 40 Na^+^ and 33 Cl^−^ ions for the TH588-MTH1 complex or the TH650-MTH1 complex. After the steepest descent minimization, each system was subjected to NVT and NPT equilibrations. During the NVT equilibration, a velocity-rescaling thermostat^29^ with the time coupling constant of 0.1 ps was conducted to remain the temperature at 300 K; in the NPT equilibration, the pressure was kept at 1 bar by coupling to the Parrinello-Rahman barostat^30^,^31^ with the time coupling constant of 2.0 ps and the isotropic compressibility of 4.5 × 10^−5^. Periodic boundary conditions were used to avoid edge effects. The bond lengths were restrained via the LINCS method^32^ to allow an integration step length of 2 fs. The particle-mesh Ewald (PME) algorithm^33^ was applied to calculate the long-range electrostatic interactions, while a cut-off radius of 1.4 nm was employed for van der Waals interactions. Finally, 500 ns molecular dynamics simulation was performed for each system in NPT ensemble.

Comparatively, the complex of MTH1 bound with 8-oxo-dGTP was also studied by molecular dynamics simulation. Briefly, the crystal structure of MTH1 bound with 8-oxo-dGTP (PDB code 5FSI^34^) was subjected to energy minimization using the chiron^35^ tool (http://troll.med.unc.edu/chiron/login.php) and then a 100 ns molecular dynamics simulation was performed. The TH287-MTH1 complex, the TH588-MTH1 complex and the TH650-MTH1 complex were also subjected to energy minimization using the chiron tool and then another independent 100 ns molecular dynamics simulations were carried out. To validate the reliability of our simulations, the free energy landscape constructed by single long simulation and multiple short simulations(ten 25-ns molecular dynamics simulations) were compared for the TH287-MTH1 complex, the TH588-MTH1 complex and the TH650-MTH1 complex.

Principle component analysis (PCA)^36^,^37^,^38^ was performed to explore the collective motions of protein or complex by addressing the positional covariance matrix *C*. The elements of this 3 N×3 N matrix *C* are defined according to Equation (1):


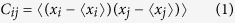


where *x*_*i*_ (*x*_*j*_) is the Cartesian coordinate of the *i*th (*j*th) C_α_ atom, 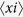 or 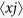 denotes the trajectory average, and N represents the number of C_α_ atom. The eigenvectors (namely the principal component) and the corresponding eigenvalues are produced by the diagonalization of covariance matrix *C*. The eigenvectors stand for the directions of atomic motions which are independent to each other in the multidimensional space, while the eigenvalues describe the corresponding magnitude.

The free energy landscape along the first two principal components (PC1 and PC2) is built by Δ*G* = −*k*_B_*T*[ln *P(V*) − ln *P_min_*], where *k*_B_ is the Boltzmann constant, T is the temperature, *P(V)* represents the probability distribution obtained from the trajectory, and *P*_*min*_ is the minimum probability distribution^39^,^40^,^41^,^42^.

The C_α_ dynamic cross correlation matrices are calculated to reveal the correlative motion of protein^43^,^44^. The elements of this matrix *S*_*ij*_ are computed by the following Equation (2):


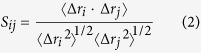


∆*r*_*i*_ and ∆*r*_*j*_ are the displacement vectors of atom i and atom j, 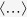 means the trajectory average. The value of *S*_*ij*_ ranges from −1 to 1. Positive value implies correlated motion, whereas negative value denotes anti-correlated motion.

## Results and Discussion

### The Movements of Compounds in the Binding Pocket Determine the Activity Difference

**Figure 2:**
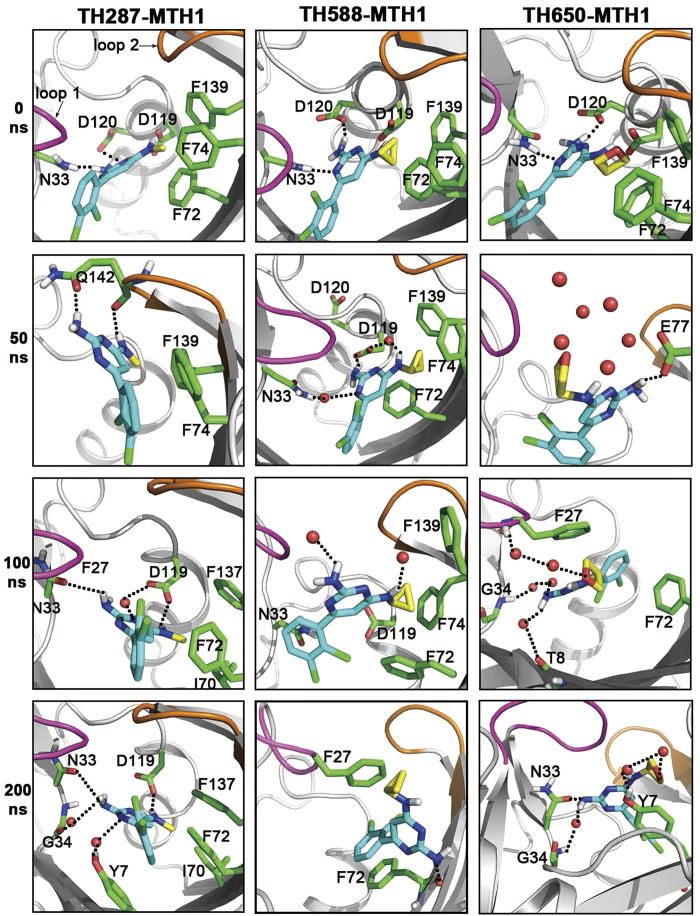
The structural characteristics of TH287-MTH1, TH588-MTH1 and TH650-MTH1 complexes from 0 ns to 200 ns. The carbon atoms of the methyl substituent of TH287, the cyclopropyl substituent of TH588 and the oxetanyl ring of TH650 are highlighted in yellow. Loop 1 (residues 23–29) and loop 2 (residues 139–143) located in the lids of the binding pocket are also highlighted.

Note that the only difference among the three compounds is that the methyl group in TH287 is replaced by the cyclopropyl group in TH588 and by the oxetanyl ring in TH650. For the starting structure, the methyl substituent of TH287 lies in the hydrophobic cavity consisting of Phe72, Phe74 and Phe139 (Fig. 2). In addition, the aminomethyl moiety of TH287 forms a hydrogen bond with the carboxyl of Asp119. The aminopyrimidine group of TH287 is involved in several significant hydrogen bonds with the side chains of Asn33, Asp119 and Asp120. As reported, 8-oxo-dGTP is supposed to recognize MTH1 by making important hydrogen bonds to Asn33, Asp119 and Asp120^18^,^34^. These suggest TH287 could occupy the recognition site of 8-oxodGTP in MTH1, and thus the oxidized nucleotides 8-oxo-dGTP could not be degraded by MTH1 and would incorporate in DNA, causing the death of cancer cell. The interactions of TH588 or TH650 with MTH1 in the starting structure are similar to TH287 (Fig. 2). That is, the cyclopropyl group of TH588 or the oxetanyl ring of TH650 also stays in the hydrophobic cavity composed of Phe72, Phe74 and Phe139. Besides, TH588 or TH650 makes key hydrogen bonds to the side chains of Asn33, Asp119 and Asp120.

In the following 50 ns, TH287 loses the hydrogen bonds with Asn33, Asp119 and Asp120. Instead, TH287 forms new hydrogen bond with Gln142 (Fig. 2). Meanwhile, the methyl group of TH287 rotates down and points to Phe139 due to its small steric hindrance. Besides, the dichlorophenyl ring of TH287 switches outside to form a π-π stacking interaction with the phenyl ring of Phe74. With regard to TH588, the steric effect of the cyclopropyl group repulses the surrounding residues including Phe72, Phe74 and Phe139, and Th588 simultaneously moves upward a bit (Fig. 2). TH588 still interacts with Asn33 and Asp119 through direct or water-mediated hydrogen bonds. However, the hydrogen bond between the aminopyrimidine group of TH588 and Asp120 is broken. TH650 also loses the hydrogen bonds with Asn33, Asp119 and Asp120. Meanwhile, the oxetanyl ring of TH650 shifts to make hydrogen bonds with the surrounding water, and the aminopyrimidine group of TH650 H-bonds to the carboxyl group of Glu77 (Fig. 2). The comparison among the three cases shows that in the first 50 ns TH287 and TH650 lose the hydrogen bonds with Asn33, Asp119 and Asp120 while TH588 keeps direct or water-mediated hydrogen bonds with Asn33 and Asp119.

During the process of 50–100 ns, because the methyl group of TH287 has a small steric effect, it can move deeply into the hydrophobic environment consisting of Ile70, Phe72 and Phe137 (Fig. 2). At the same time, TH287 recovers the direct or water-mediated hydrogen bonds with key residues Asn33 and Asp119. For the case of TH588, the cyclopropyl group continues repulsing the surrounding residues (Phe72, Phe74 and Phe139), which may facilitate the aminocyclopropyl and aminopyrimidine groups of TH588 rotate to contact with the solvent and lose the significant polar interactions with Asn33 and Asp119 (Fig. 2). Different from TH287 and TH588, two water molecules come from outside to mediate the interaction between the oxetanyl ring of TH650 and the amidogen of Phe27 which is located in the lid of binding pocket (Fig. 2). Meanwhile, the aminopyrimidine group of TH650 rotates from the solvent interface to the binding pocket driven by the water-mediated hydrogen bonds to Gly34 and Thr8. Additionally, the dichlorophenyl ring of Th650 approaches Phe72 to form T-shaped stacking. Apparently, in the process of 50–100 ns, the small steric effect of the methyl substituent permits TH287 recovers the key hydrogen bonds with Asn33 and Asp119, whereas TH588 and TH650 cannot recover hydrogen bonds with Asn33 and Asp119 due to the large steric hindrances of the cyclopropyl substituent in TH588 and the oxetanyl substituent in TH650.

In the 100–200 ns step, the methyl substituent of TH287 steadily stays in the hydrophobic pocket composed of Ile70, Phe72 and Phe137 due to its slight steric hindrance (Fig. 2). The aminomethyl and aminopyrimidine groups of TH287 keep significant hydrogen bonds or water-mediated interactions with Asn33 and Asp119. One different feature from the previous 100 ns is that two waters mediate the pyrimidine moiety to interact with Gly34 and Tyr7. In addition, the dichlorophenyl ring of TH287 forms a sandwich structure together with Phe72 and Tyr7. Unlike TH287, the cyclopropyl substituent of TH588 leaves the hydrophobic pocket (Phe72, Phe74 and Phe139), and then move up to contact with Phe27 which locates at the lid of the binding pocket (Fig. 2). The aminopyrimidine group of TH588 turns from the solvent interface to the binding site, driven by the formation of the hydrogen bond between its aminopyrimidine group and the carbonyl group of Phe72. In addition, the dichlorophenyl ring of TH588 π-stacks with Phe72. For the TH650-MTH1 complex, due to the flow of solvent, the water bridge connecting the oxetanyl ring of TH650 with Phe27 is broken, and two new waters mediate the interaction between the oxetanyl ring and the hydroxyl of Tyr7 (Fig. 2). The aminopyrimidine group of TH650 still keeps water-mediated hydrogen bond with Gly34, but the water-mediated hydrogen bond with Thr8 is broken and the new hydrogen bonds with Asn33 forms. The comparison among the three complexes demonstrates that during the 100–200 ns process, the methyl substituent of TH287 steadily stays in the binding site, whereas the cyclopropyl substituent of TH588 move up to the margin of the binding pocket and the oxetanyl substituent of TH650 point toward the solvent environment.

In the following 200–500 ns process, the methyl substituent of TH287 stably lies in the hydrophobic pocket composed of I70, F72 and F137, and the aminomethyl and aminopyrimidine groups of TH287 maintain hydrogen bonds with Asn33 and Asp119 in the binding site (Figure S1 in the Supplementary Information (SI)). For the case of TH588-MTH1 complex, the cyclopropyl substituent of TH588 maintains the hydrophobic interaction with Phe27, the aminopyrimidine group keeps stable hydrogen bonds with the carbonyl groups of Phe72 and Met81, and the dichlorophenyl ring π-stacks with Phe72 (Figure S1 in the SI). As reported, Phe72 is very important for MTH1 to recognize ligands^16^. Interestingly, TH588 also forms stable interaction with Phe72, explaining why TH588 has relatively high potency to inhibit MTH1. For the case of TH650-MTH1 complex, the oxetanyl substituent of TH650 tends to interact with the solvent (Figure S1 in the SI). The flow of solvent may promote TH650 undergo more significant movement in the binding pocket than TH287 and Th588. Besides, TH650 occasionally form direct or water-mediated hydrogen bonds with Val75 and Phe27, Asn33 and Lys23 (Figure S1 in the SI). However, these hydrogen bonds are less stable than those between MTH1 and TH287 or Th588. Apparently, during the 200–500 ns process, TH287 stays stably in the binding site through H-bonding to Asn33 and Asp119, which may be responsible for the high inhibitory activity of TH287. TH588 loses significant hydrogen bonds with Asn33 and Asp119 but forms hydrogen bonds with Phe72 and Met81 and π-π stacking with Phe72. Thus, TH588 has a slightly reduced inhibitory activity compared to TH287. By contrast, TH650 cannot stably lie in the binding site due to the interference of solvent on the oxetanyl substituent of TH650, responsible for the lowest inhibitory activity of TH650.

In order to further explore the binding status of ligands, the distances between ligands and key residues as a function of time are calculated. Because Asn33 and Asp119 of MTH1 are very significant to recognize the oxidized nucleotides^7^, the distances between ligands and Asn33 or Asp119 are emphasized. The TH287-N4__Asn33-OD1 distance keeps about 3.5 Å from 100 ns to 500 ns, while the TH287-N3__Asp119-OD1 distance maintains about 3.0 Å during the process of 100–350 ns (Figure S2 in the SI), suggesting that TH287 makes significant hydrogen bonds with Asn33 and Asp119 in most of the simulation time. Therefore, TH287 has high inhibitory activity against MTH1. The TH588-N1__Asn33-ND2 distance and the TH588-N4__Asp119-OD1 distance just keep ~3.5 Å in the first 40 ns, indicating that TH588 is unable to make stable hydrogen bonds with Asn33 and Asp119. This may contribute to the lower inhibitory activity of TH588 than TH287. The possible reason is that the cyclopropyl substituent of TH588 has larger steric hindrance than the methyl substituent of TH287. However, the TH588-N4__Phe72-O distance keeps about 3.0 Å from 110 ns to 500 ns, while the TH588-N4__Met81-N distance maintains about 3.5 Å during the process of 110–500 ns (Figure S2 in the SI), implying that TH588 can make stable hydrogen bonds with Phe72 and Met81. Besides, the distance between the dichlorophenyl ring of TH588 and the phenyl ring of Phe72 fluctuates around 5 Å from 110 ns to 500 ns, suggesting that TH588 can form π-π stacking with Phe72. Therefore, the stable interactions of TH588 with Phe72 and Met81 may contribute to the relatively high inhibitory activity of TH588 against MTH1. The molecular dynamics simulations performed by Niu *et al*. suggested that Phe72 was very important for the selective binding of inhibitor to MTH1^16^, supporting the interaction of TH588 with Phe72 could facilitate the binding of TH588 to MTH1. By contrast, the TH650-N4__Asn33-OD1 distance decreases to ~3.0 Å in the last 100 ns, while the TH650-N4__Asp119-OD1 distance is almost larger than 3.5 Å in the whole simulation (Figure S2 in the SI), indicating that TH650 is unable to form such stable interactions with Asn33 and Asp119 as TH287. These data may contribute to the lower inhibitory activity of TH650 than TH287. A comparison between TH650 and TH588 shows that TH650 cannot form stable hydrogen bonds during the simulation while TH588 can make stable hydrogen bonds with Phe72 and Met81. Thus, the inhibitory activity of TH650 is lower than TH588. There are some possible reasons responsible for the low inhibitory activity of TH650. For one thing, the oxetanyl substituent of TH650 has larger steric hindrance than TH287 and TH588. For another, the oxetanyl substituent of TH650 makes polar interactions with the solvent, and the flow of the solvent prevents TH650 forming stable interactions with MTH1. Clearly, the distances between ligands and key residues as a function of time help to illuminate the reasons why the inhibitory activity follows a TH287 > TH588 > TH650 trend.

### Hydrogen-bond Probability Causes the Activity Difference of Compounds

To further explore the polar interactions between the three compounds and MTH1 protein, the occupancies of hydrogen bonds are analyzed (Table 1, 2 and 3). It is shown that TH287 keeps stable H-bonds with the OD1 atom of Asn33, the OD1 atom of Asp119 and the OD2 atom of Asp119, with their time occupancies of 61%, 49% and 25%, respectively. Furthermore, 8-oxo-dGTP also maintains stable H-bonds with the OD1 atom of Asn33, the OD1 atom of Asp119 and the OD2 atom of Asp119, with the occupancies of 65%, 96% and 85%, respectively. Apparently, TH287 and 8-oxo-dGTP have similar hydrogen-bond interactions with MTH1, and thus TH287 could well occupy the active site of the oxidized nucleoside triphosphate in MTH1, which may be the key reason that TH287 has high inhibitory activity against MTH1. By contrast, TH588 is unable to keep stable hydrogen bonds with significant residues Asn33 and Asp119 with the occupancies of 8% and 10%, leading to the inhibitory activity of TH588 lower than TH287. However, TH588 maintains stable hydrogen bonds with Phe72 and Met81 with the occupancies of 77% and 73%. These stable hydrogen bonds promote TH588 occupies the binding pocket of MTH1, and thus TH588 presents the relatively high bioactivity. On the contrary, TH650 forms hydrogen bonds to Asn33 and Asp119 with the low occupancies of 30% and 1.8%. Besides, all the probabilities of TH650 H-bonding to MTH1 are lower than 30%. These data suggest that TH287 and TH588 have more stable hydrogen bonds than TH650, which is in accordance with the experimental phenomenon that TH287 and TH588 have higher inhibitory activities than TH650.

### Compounds Change the Flexibility of MTH1 Leading to the Activity Difference

**Figure 3:**
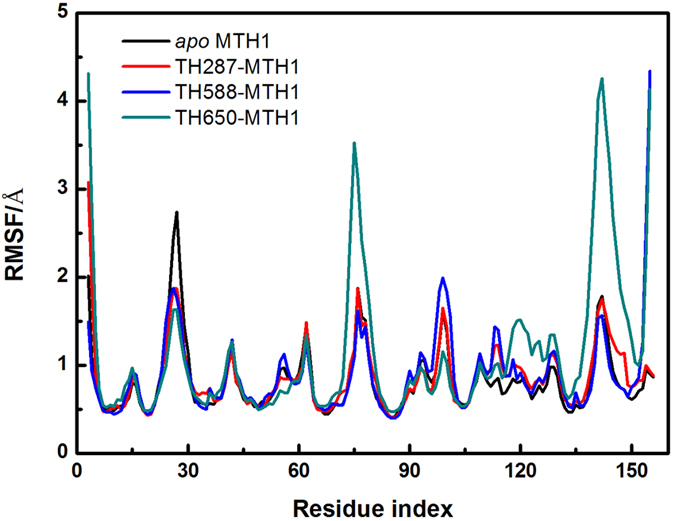
RMSF of backbone atoms for the *apo* MTH1, TH287-MTH1 complex, TH588-MTH1 complex and TH650-MTH1 complex.

The root mean squared deviations (RMSF) values of residues 23–29, residues 75–79 and residues 139–143 of the *apo* MTH1 reach up to ~2.7 Å, ~1.9 Å and ~1.8 Å (Fig. 3), which are higher than other regions, suggesting that these three loops are most flexible. Resides 23–29 of the TH287-MTH1 complex (RMSF 1.0–1.9 Å) are more rigid than those of the *apo* MTH1 (RMSF 0.9–2.7 Å), indicating that TH287 can stabilize the conformation of residues 23–29. This is because the aminopyrimidine moiety of TH287 can form H-bond network with Lys23 and Phe27 via water bridges (Figure S3 in the SI). RMSF values of resides 23–29 of the TH588-MTH1 complex are also lower than the counterpart of the *apo* MTH1, implying that TH588 also make the conformation of residues 23–29 more stable. The possible reason is that the cyclopropyl of TH588 has hydrophobic interaction with Phe27 (Figure S4 in the SI). Residues 23–29 of the TH650-MTH1 complex are more rigid than the counterparts of the *apo* MTH1. This is because TH650 forms direct or water-mediated hydrogen bonds with Lys23 and Phe27 (Figure S5 in the SI). However, RMSF values of residues 75–79 and residues 139–143 of the TH650-MTH1 complex are ~2 Å and ~3 Å higher than other three cases (the *apo* MTH1, the TH287-MTH1 complex and the TH588-MTH1 complex). Obviously, TH650 can induce more remarkable conformational fluctuation of MTH1 than TH287 and TH588. This may partially explain why the activity of TH650 is much lower than TH287 and TH588.

### Compounds Induce the Opening or Closure of Binding Pocket resulting in the Activity Difference

In order to illuminate the collective motion of MTH1 induced by the three compounds, principal component analysis (PCA) was carried out. The first components (PC1), representing the most significant motions, should be emphatically analyzed.

**Figure 4:**
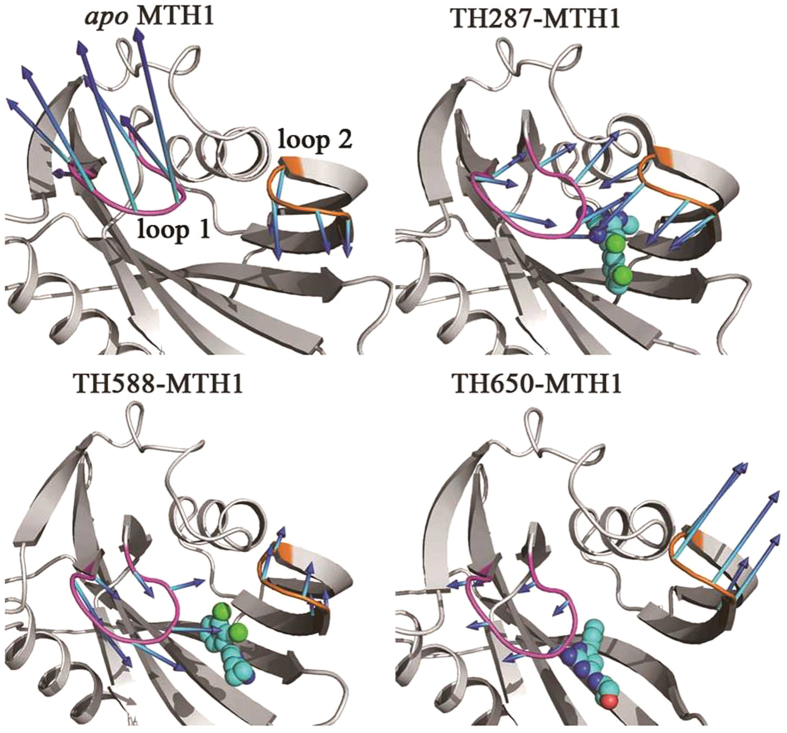
Collective motions corresponding to the PC1 for *apo* MTH1, TH287-MTH1 complex, TH588-MTH1 complex and TH650-MTH1 complex. The average structures are shown, the arrow represents the motion direction, and the arrow length describes the motion magnitude.

**Figure 5:**
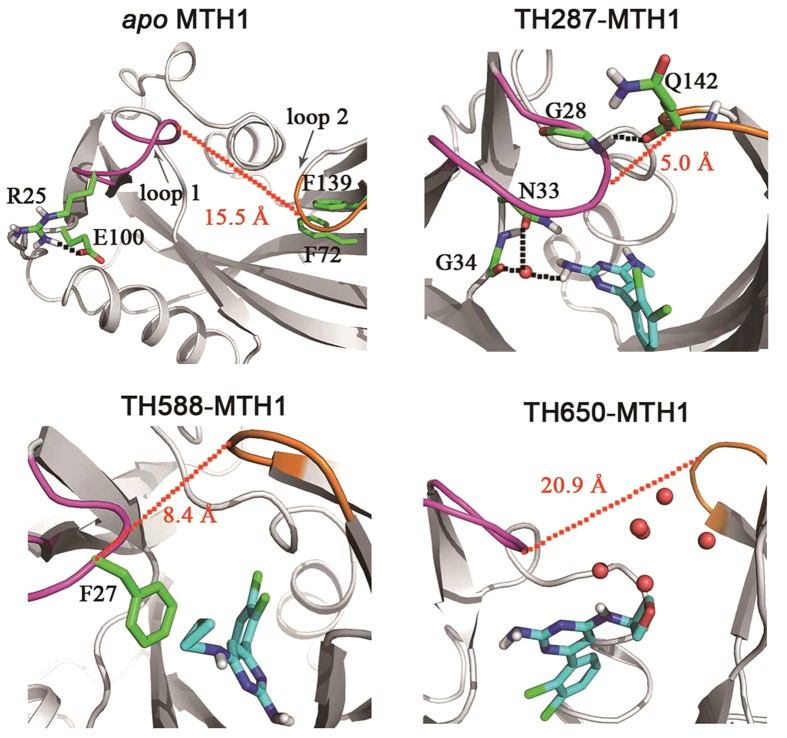
The snapshots with the maximum eigenvalues of the first components (PC1) for *apo* MTH1, TH287-MTH1 complex, TH588-MTH1 complex and TH650-MTH1 complex.

According to RMSF analysis, residues 23–29 (loop 1) and residues 139–143 (loop 2) are very flexible. Additionally, these two loops are the lids of the binding pocket. Therefore, loop 1 and loop 2 are used to characterize the opening or closure of the binding pocket. The PC1 motion of the *apo* MTH1 shows that loop 1 switches outwards from the binding pocket while loop 2 migrates inwards to the binding pocket (Fig. 4). In other words, loop 1 and loop 2 adopt an anti-parallel motion. For one thing, Arg25 of loop 1 H-bonds to Glu100 which is far away from the binding pocket, resulting in loop 1 turning outside (Fig. 5). For another, Phe139 of loop 2 π-stacks to Phe72 which locates in the binding pocket, facilitating loop 2 moving inside (Fig. 5).

In regarding to the PC1 component of the TH287-MTH1 complex, loop 1 and loop 2 adopt an approaching motion induced by TH287, resulting in the closure of the binding pocket (Fig. 4). On one hand, the aminopyrimidine moiety of TH287 forms direct or water-mediated hydrogen bonds with Asn33 and Gly34, inducing loop 1 move to the binding pocket. On the other hand, the formation of the hydrogen bond between Gly28 of loop 1 and Gln142 of loop 2 directly causes the closure of these two flexible loops (Fig. 5).

For the PC1 motion of the TH588-MTH1 complex (Fig. 4), loop 1 moves towards the binding pocket which is similar to the case of the TH287-MTH1 complex. This motion may be driven by the hydrophobic interaction between the cyclopropyl substituent of TH588 and Phe27 of loop 1 (Fig. 5). In addition, loop 2 shifts away from the binding pocket which is opposite to the situation of the TH287-MTH1 complex, which may be attributed to the larger steric hindrance of TH588 than TH287. However, in the TH588-MTH1 complex, the motion amplitude of loop 1 is larger than loop 2. Thus, the binding pocket tends to take a semi-closed state.

With respect to the PC1 component of the TH650-MTH1 complex (Fig. 4), loop 1 and loop 2 adopt the opening motion. The possible reasons may be the steric hindrance of the oxetanyl substituent and the polar disturbance of solvent phase on the oxetanyl substituent.

Furthermore, the C_α_ atom distances between Phe27 of loop 1 and Gln142 of loop 2 in the structures with the maximum eigenvalues of PC1 are 15.5 Å, 5.0 Å, 8.4 Å and 20.9 Å for the *apo* MTH1, the TH287-MTH1 complex, the TH588-MTH1 complex and the TH650-MTH1 complex, respectively (Fig. 5). These data also indicate that the TH287-MTH1 complex adopts the closed conformation, the TH588-MTH1 complex adopts the semi-closed conformation, and the TH650-MTH1 complex adopts the open conformation.

In brief, the closed state of the TH287-MTH1 complex would well restrict the dissociation of TH287, and thus TH287 has a high inhibitory activity against MTH1. The semi-closed state of the TH588-MTH1 complex could restrict the dissociation of TH588 to some extent, and thus TH588 has a slight reduced inhibitory activity. By contrast, the open state of the TH650-MTH1 complex would facilitate the dissociation of TH650, and thus TH650 has a low inhibitory activity.

### Compounds Induce Diverse Correlated Motions of MTH1 Causing the Activity Difference

**Figure 6:**
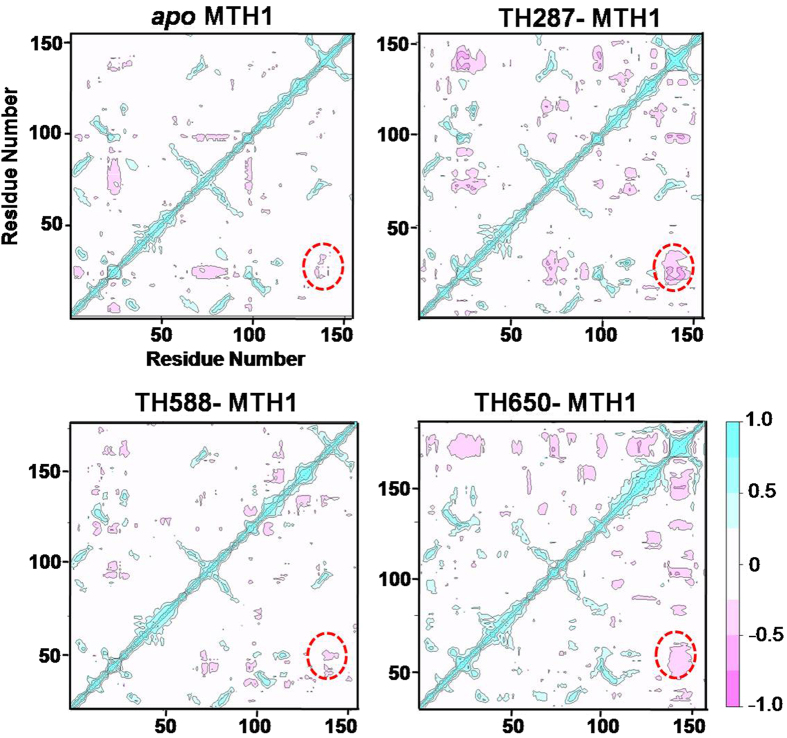
The cross-correlation matrix analysis for *apo* MTH1, TH287-MTH1 complex, TH588-MTH1 complex and TH650-MTH1 complex. The regions including loop 1 (residues 23–29) and loop 2 (residues 139–143) are highlighted in dotted circle.

In order to further reveal the effect of compounds on the correlated motion of MTH1 protein, the cross-correlation matrix analysis was carried out (Fig. 6). In the *apo* MTH1, loop 1 (residues 23–29) and loop 2 (residues 139–143) have slight anticorrelated movements. It is noted that the anticorrelated movements represent the opposite directional movements. However, TH287 can dramatically enhance the anticorrelated movements of loop 1 and loop 2, and this anticorrelated movement characterizes the closure of MTH1 according to the PCA results. The closed state of TH287-MTH1 complex would restrict the dissociation of Th287 and enhance the inhibitory activity of TH287. The anticorrelated movement of loop 1 and loop 2 in the TH588-MTH1 complex is weaker than the TH287-MTH1 complex, which corresponds to the semi-closure of MTH1 in the PCA analysis. Thus, the semi-closed state of TH588-MTH1 complex has lower capacity to restrict the dissociation of ligand than the closed state of TH287-MTH1 complex. This may help to explain why the bioactivity of TH588 is lower than TH287. TH650 can strengthen the anticorrelated movements of loop 1 (residues 23–29) and loop 2 (residues 139–143), and this anticorrelated movement in TH650-MTH1 complex corresponds to the opening of MTH1 according to the PCA results. The open state of the TH650-MTH1 complex would facilitate the dissociation of TH650 and reduce the bioactivity of TH650. In a word, the cross-correlation matrix results are consistent with PCA results, both of which help to reveal why the bioactivity of compounds against MTH1 follows a TH287 > TH588 > TH650 trend.

### The Free Energy Landscapes Clarify the Activity Difference

**Figure 7:**
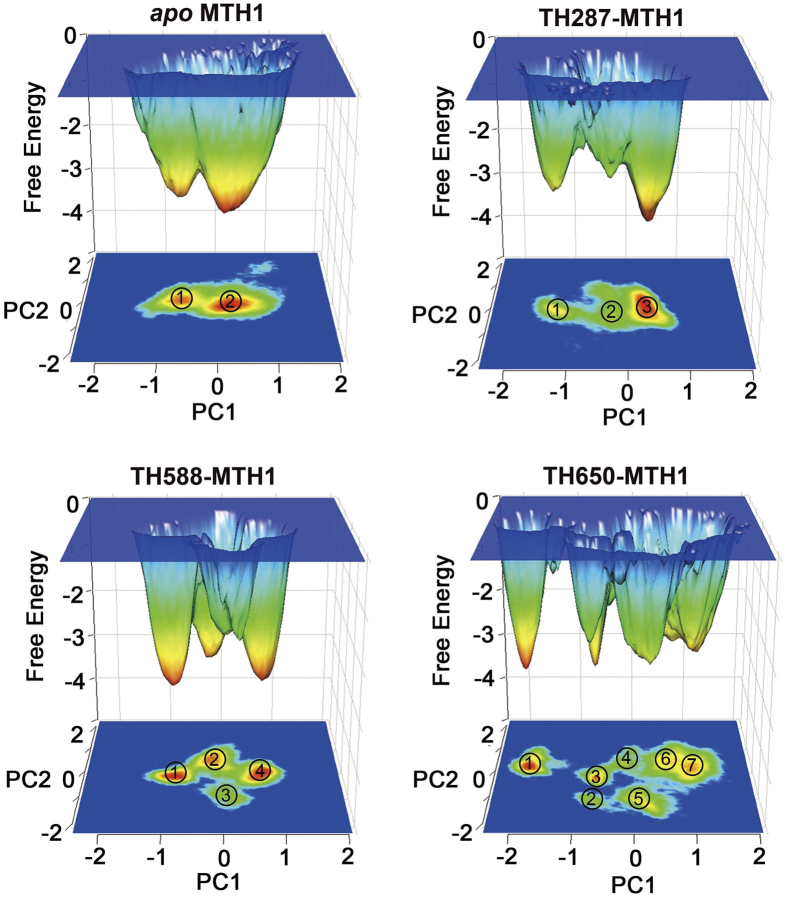
Free Energy landscapes of the *apo* MTH1, TH287-MTH1 complex, TH588-MTH1 complex and TH650-MTH1 complex.

To further explore the conformational space of MTH1 stabilized by the three compounds, the probability-based free energy landscapes as a function of the first two principal components (PC1 and PC2) were comparatively analyzed (Fig. 7). From the constructed free energy landscapes, two major ensembles of conformations are identified for the *apo* MTH1, while three major ensembles of conformations are identified for the MTH1 protein bound with TH287, showing that TH287 can induce the conformational change of MTH1. However, the MTH1 protein bound with TH287 has concentrated conformational space which is similar to the *apo* MTH1. The MTH1 protein in the presence of TH588 has four major conformation ensembles. This demonstrates TH588 can induce one more conformation ensemble than TH287, showing TH588 may have lower ability to stabilize MTH1 protein than TH287 and thus TH588 has lower bioactivity than TH287. Even so, the localization of the ensembles stabilized by TH588 is also concentrated, which may be related to the relatively high activity of TH588. In contrast, the conformational space of MTH1 stabilized by TH650 involves seven ensembles. This suggests TH650 can induce four more conformation ensembles than TH287 and three more conformation ensembles than TH588, showing TH650 has lowest capacity to stabilize MTH1 protein and thus TH650 has lowest inhibitory activity. Additionally, the localization of the ensembles stabilized by TH650 is much more discrete than those stabilized by TH287 and TH588, which may be related to the lowest bioactivity of TH650. In brief, the free energy landscapes reveal that the ability of compounds stabilizing the MTH1 protein follows a TH287 > TH588 > TH650 trend, which well matches with the pharmacological properties of the compounds. As shown in the free energy landscapes extracted from other independent 100 ns simulations (Figure S6 in the SI) and from multiple short simulations (Figure S7 in the SI), the numbers of major ensembles of the TH287-MTH1complex is less than the TH588-MTH1 complex while the numbers of major ensembles of the TH588-MTH1complex is less than the TH650-MTH1complex, reproducing that the ability of compounds stabilizing the MTH1 protein follows a TH287 > TH588 > TH650 trend and also supporting the reliability of our simulations.

## Conclusion

In this study, molecular dynamics simulations were performed to reveal the mechanisms why TH287, TH588 and TH650 have different inhibitory activities against MTH1. Several conclusions were drawn as follows.

The movements of compounds in the binding pocket elucidate that TH287 stably stays in the binding site and H-bonds to Asn33 and Asp119 owing to the small steric hindrance of its methyl substituent, which may be responsible for the high inhibitory activity of TH287 against MTH1. Because the cyclopropyl substituent of TH588 has larger steric hindrance than the methyl substituent of TH287, TH588 loses significant hydrogen bonds with Asn33 and Asp119 but forms hydrogen bonds with Phe72 and Met81 and π-π stacking with Phe72. Thus, TH588 has a slightly reduced inhibitory activity compared to TH287. By contrast, TH650 cannot stably lie in the binding site because of not only the large steric hindrance of the oxetanyl substituent but also the interference of solvent on the oxetanyl substituent, being responsible for the low inhibitory activity of TH650.

The hydrogen bond analysis indicates that TH287 keeps hydrogen bonds with significant residues Asn33 and Asp119 with the time occupancies of 61% and 49%, and thus TH287 has high inhibitory activity against MTH1. TH588 is unable to keep stable hydrogen bonds with Asn33 and Asp119 with the occupancies of 8% and 10%, but TH588 maintains stable hydrogen bonds with Phe72 and Met81 with the occupancies of 77% and 73%, leading to the medium inhibitory activity of TH588. TH650 forms unstable hydrogen bonds to Asn33 and Asp119 with low occupancies of 30% and 1.8%, resulting in the low inhibitory activity of TH650.

RMSF analysis reveals that RMSF of residues 75–79 and residues 139–143 of the TH650-MTH1 complex being 2–3 Å higher than the counterparts of the TH287-MTH1 and TH588-MTH1 complexes, suggesting that TH287 and TH588 can stabilize the conformation of MTH1 while TH650 can induce larger conformational fluctuation of MTH1.

According to PCA and cross-correlation matrix analysis, TH287 induces MTH1 protein adopt a closure motion, TH588 promotes MTH1 adopt a semi-closed motion, and TH650 induces MTH1 adopt an opening motion.

Based on the free energy landscape analysis, the number of conformational ensembles follows the trend of the TH288-MTH1 complex <the TH588-MTH1 complex <the TH650-MTH1 complex, showing the ability of compounds stabilizing the MTH1 protein follows a TH287 > TH588 > TH650 trend.

Above all, the inhibitory activity follows a TH287 > TH588 > TH650 trend, which well matches with the experimental finding. Our results may provide some cues to design new inhibitors of MTH1.

## Additional Information

**How to cite this article**: Wang, M. *et al*. Understanding the molecular mechanism for the differential inhibitory activities of compounds against MTH1. *Sci. Rep.* **7**, 40557; doi: 10.1038/srep40557 (2017).

**Publisher's note:** Springer Nature remains neutral with regard to jurisdictional claims in published maps and institutional affiliations.

## Supplementary Material

Supplementary Information

## Figures and Tables

**Figure 1:**
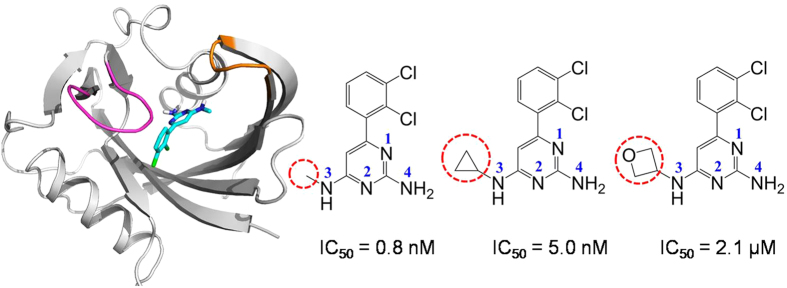
The co-crystal structure of MTH1 bound with TH287, and the chemical structures of TH287, TH588 and TH650. The differences of the three compounds are highlighted in dotted circle. Each nitrogen atom of ligands is labeled to avoid the confusion.

**Table 1:** Hydrogen bond occupancy involving interactions between TH287 and MTH1.

Ligand (TH287)	Protein	Hydrogen bond occupancy
TH287-N4	Asn33 -OD1	61%
TH287-N3	Asp119 -OD1	49%
TH287-N3	Trp123-NE1	30%
TH287-N3	Asp119-OD2	25%
TH287-N4	Asn33-ND2	18%
TH287-N4	Trp117 -NE1	15%
TH287-N3	Gln142-O	15%

**Table 2:** Hydrogen bond occupancy involving interactions between TH588 and MTH1.

Ligand (TH588)	Protein	Hydrogen bond occupancy
TH588-N4	Phe72-O	77%
TH588-N4	Met81-N	73%
TH588-N4	Glu79-O	71%
TH588-N4	Phe74-N	56%
TH588-N4	Glu73-N	38%
TH588-N4	Asp119-OD1	10%
TH588-N1	Asn33-ND2	8%

**Table 3:** Hydrogen bond occupancy involving interactions between TH650 and MTH1.

Ligand (TH650)	Protein	Hydrogen bond occupancy
TH650-N4	Asn33-OD1	30%
TH650-N4	Lys23-NZ	29%
TH650-O	Val75 -N	21%
TH650-N3	Tyr7-OH	20%
TH650-N2	Tyr7-OH	18%
TH650-N4	Asp119 -OD1	1.8%
