# Supplementary material for: Understanding the molecular mechanism for the differential inhibitory activities of compounds against MTH1
Source: Sci Rep. 2017 Jan 11;7:40557. doi: 10.1038/srep40557 (PMC5225434; doi:10.1038/srep40557)
Supplement: Supplementary Information [file srep40557-s1.pdf]

Supplementary Information

Understanding the molecular mechanism for the differential inhibitory activities of compounds against MTH1

Mian Wang[1,2,3], Shuilian Zhou[3], Qing Chen[3], Lisheng Wang[3], Zhiqun Liang[1,2]*, Jianyi Wang[3]*

1 *State Key Laboratory for Conservation and Utilization of Subtropical Agro-Bioresources, Guangxi University, Nanning 530004, People's Republic of China,*

2 *College of Life Science and Technology, Guangxi University, Nanning 530004, People's Republic of China,*

3 *School of Chemistry and Chemical Engineering, Guangxi University, Nanning 530004, People's Republic of China*

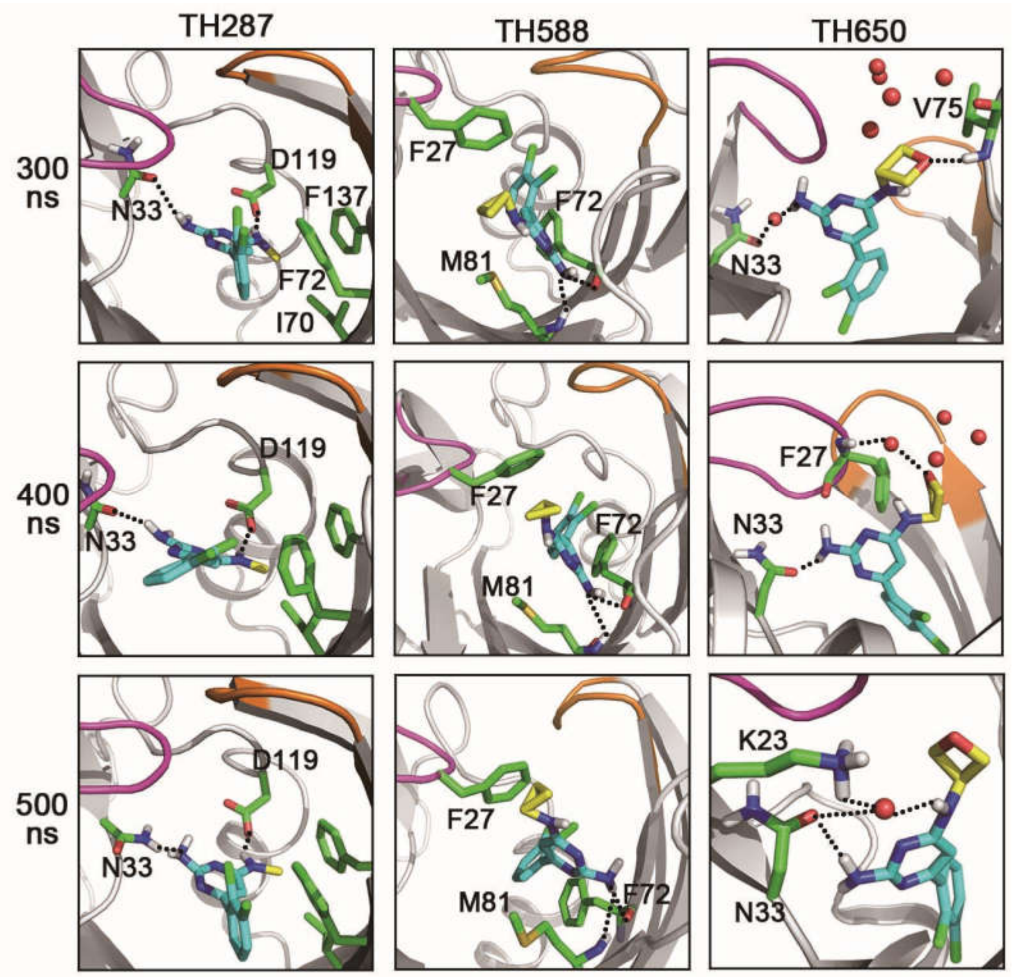

Figure S1. The structural characteristics of the TH287-MTH1, TH588-MTH1 and TH650-MTH1 complexes from 300 ns to 500 ns.

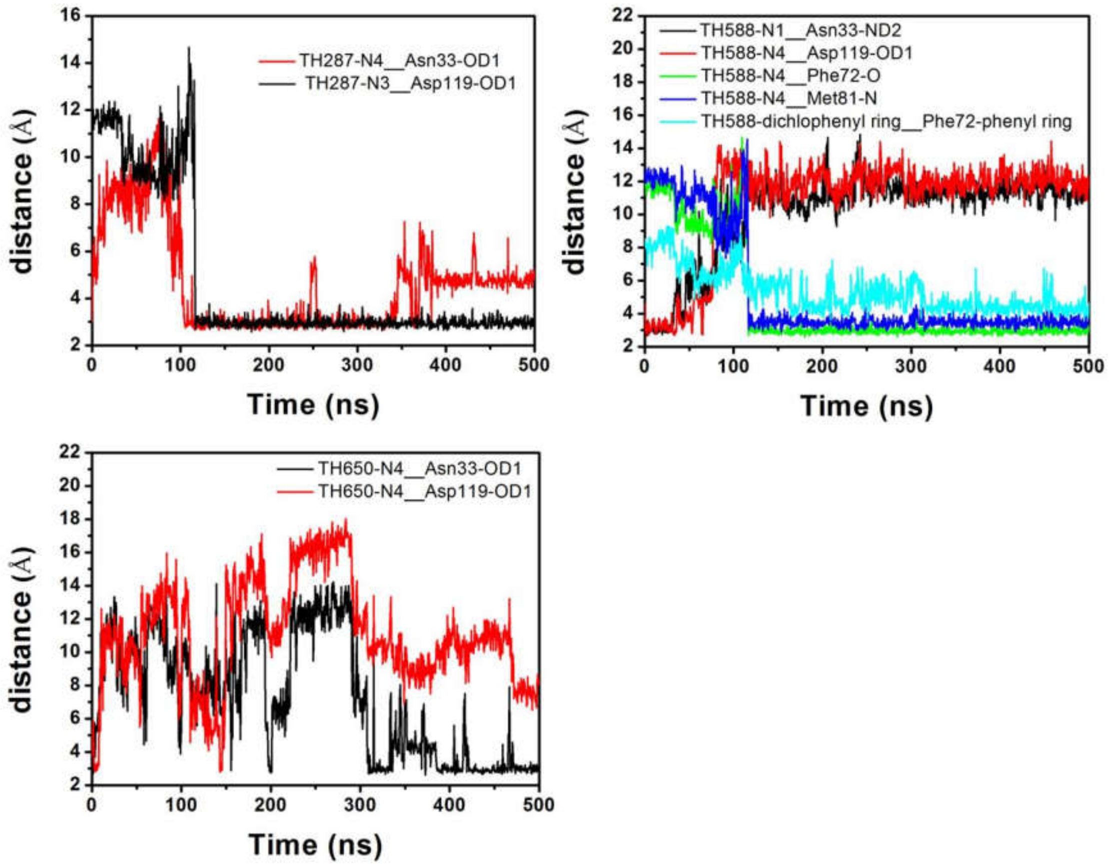

Figure S2. The distances between ligands and concerned residues as a function of time.

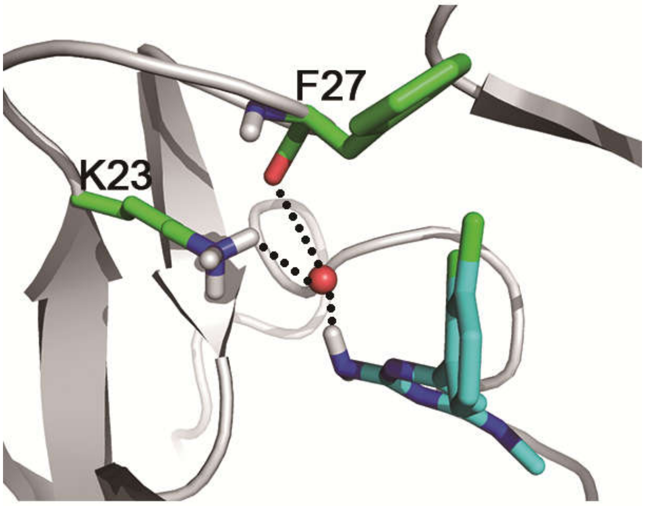

Figure S3. The typical snapshot of the TH287-MTH1 complex at 300 ns.

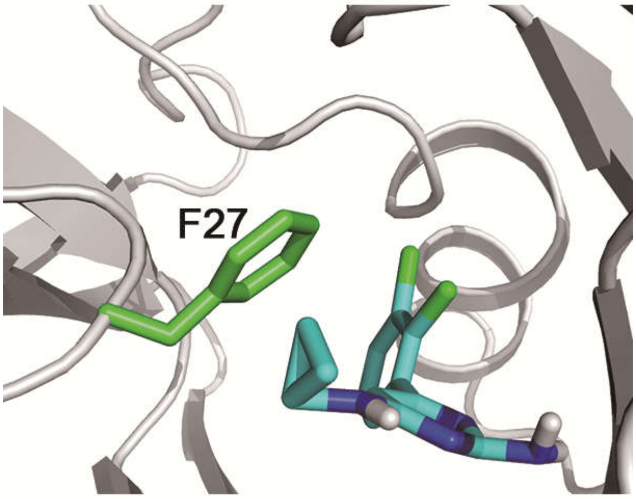

Figure S4. The typical snapshot of the TH588-MTH1 complex at 500 ns.

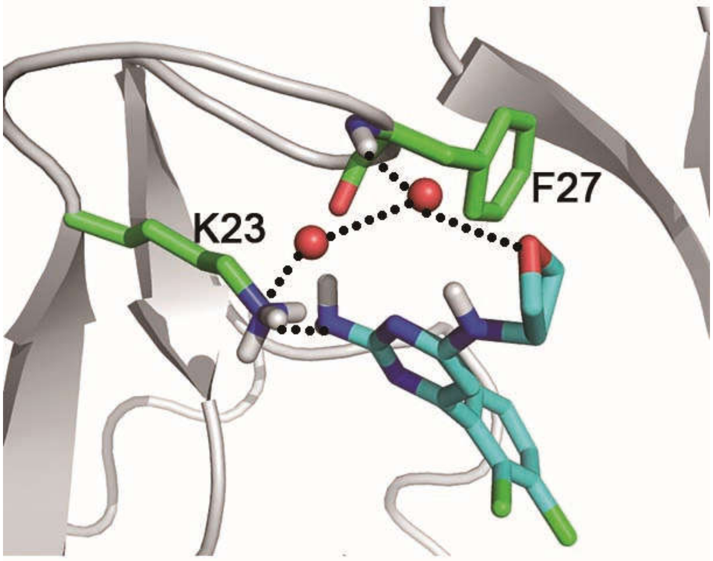

Figure S5. The typical snapshot of the TH650-MTH1 complex at 400 ns.

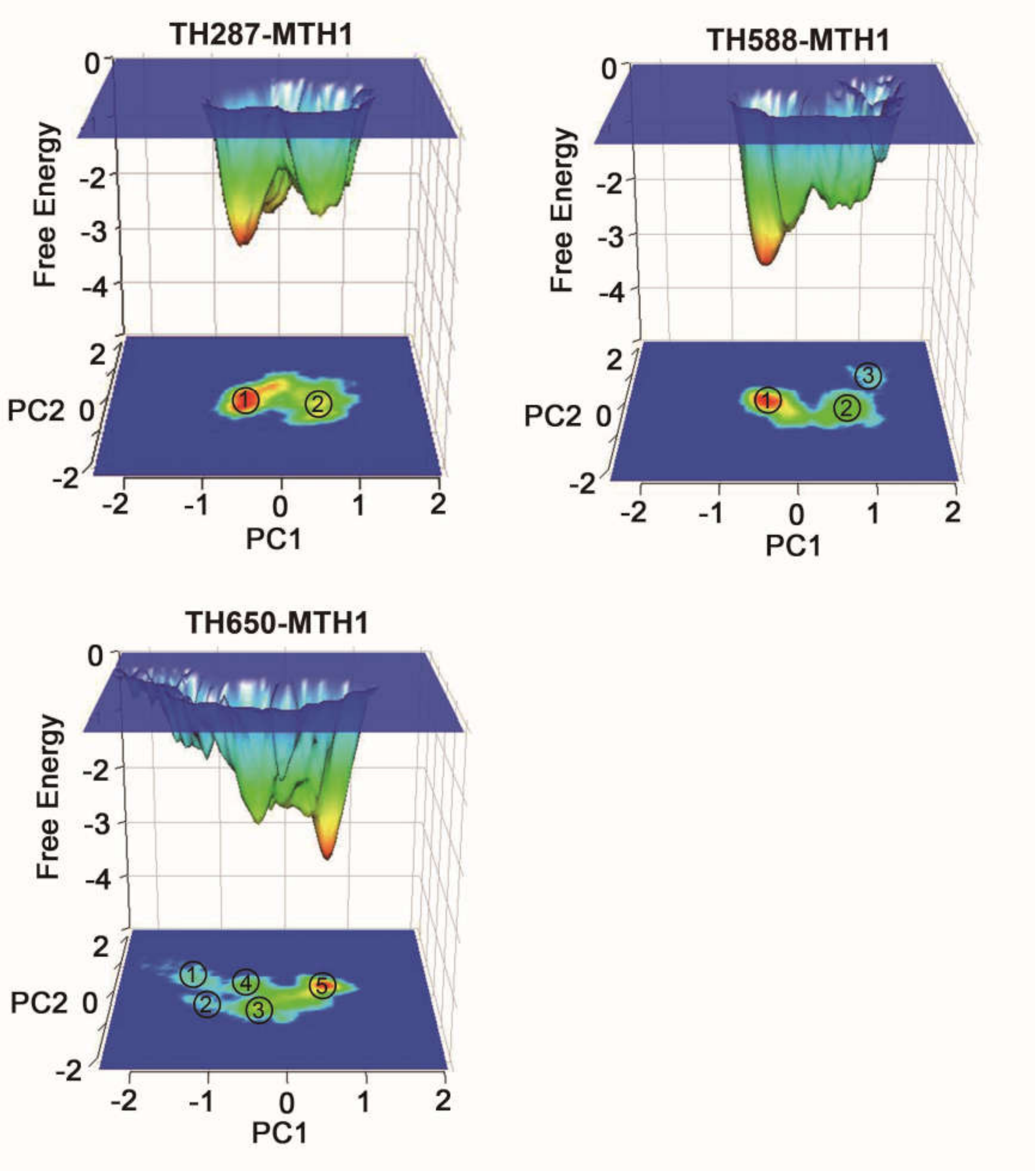

Figure S6. Free Energy landscapes of the TH287-MTH1 complex, TH588-MTH1 complex and TH650-MTH1 complex for another independent 100 ns simulations.

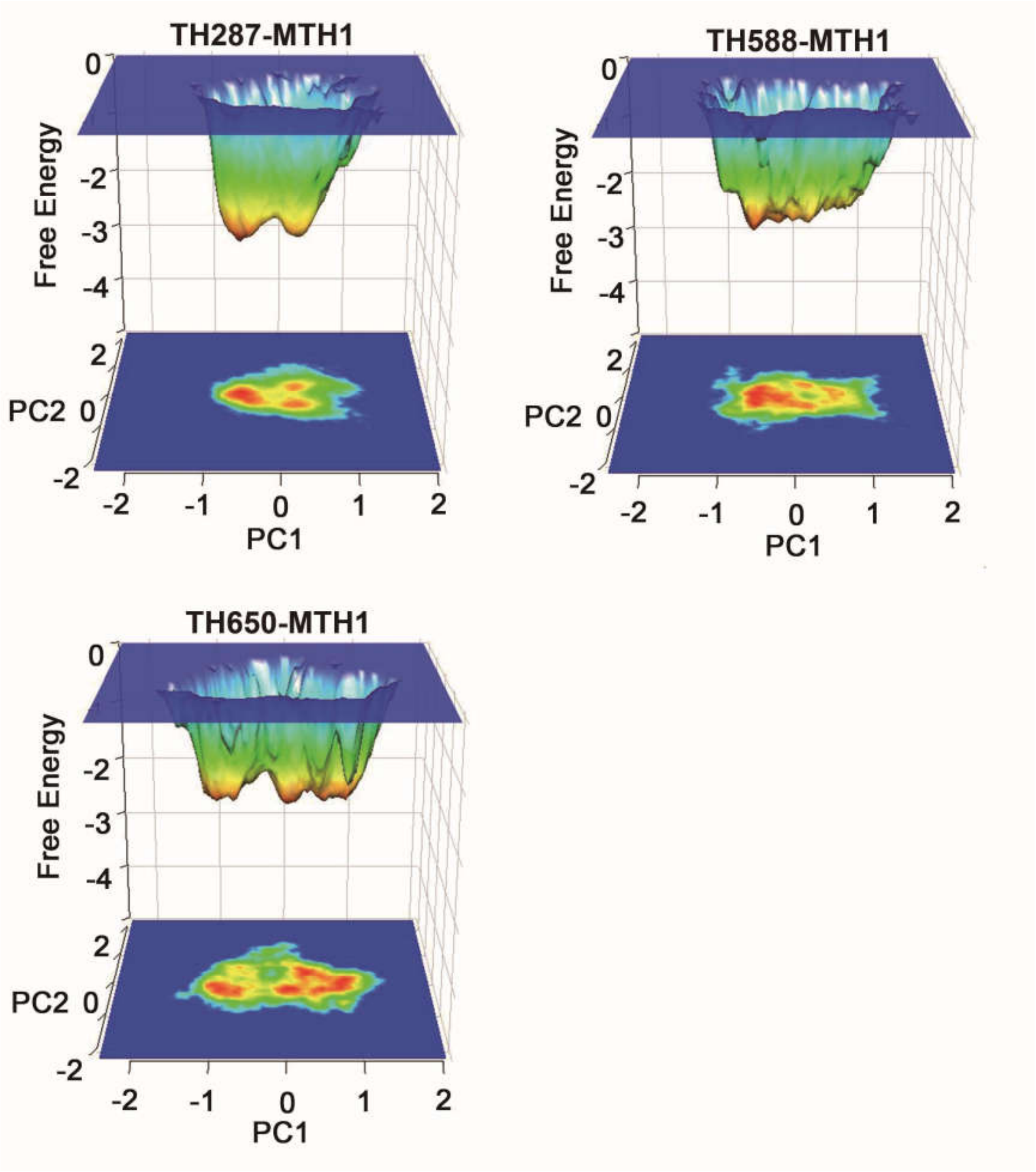

Figure S7. Free Energy landscapes of the TH287-MTH1 complex, TH588-MTH1 complex and TH650-MTH1 complex for multiple short simulations.

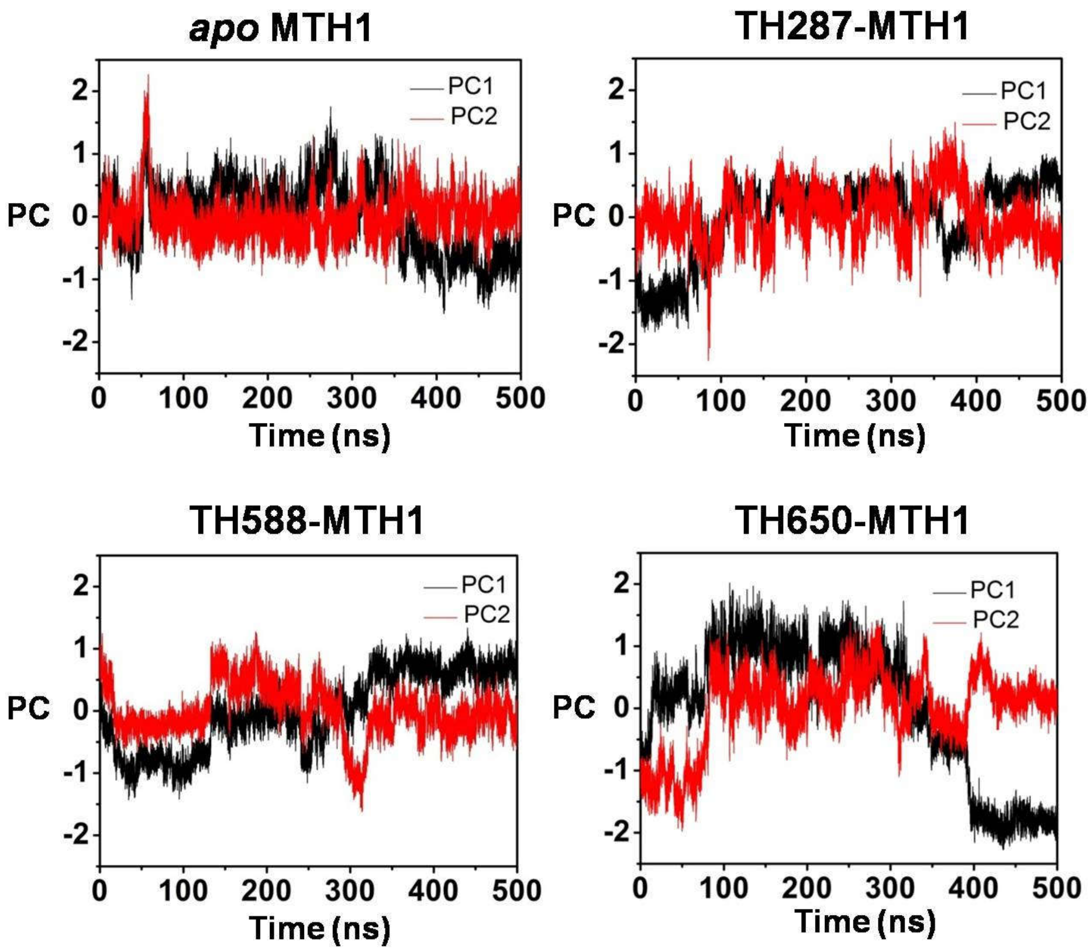

Figure S8. Time evolutions of the first two principal components (PC1 and PC2) for the *apo* MTH1, the TH287-MTH1 complex, the TH588-MTH1 complex and the TH650-MTH1 complex.

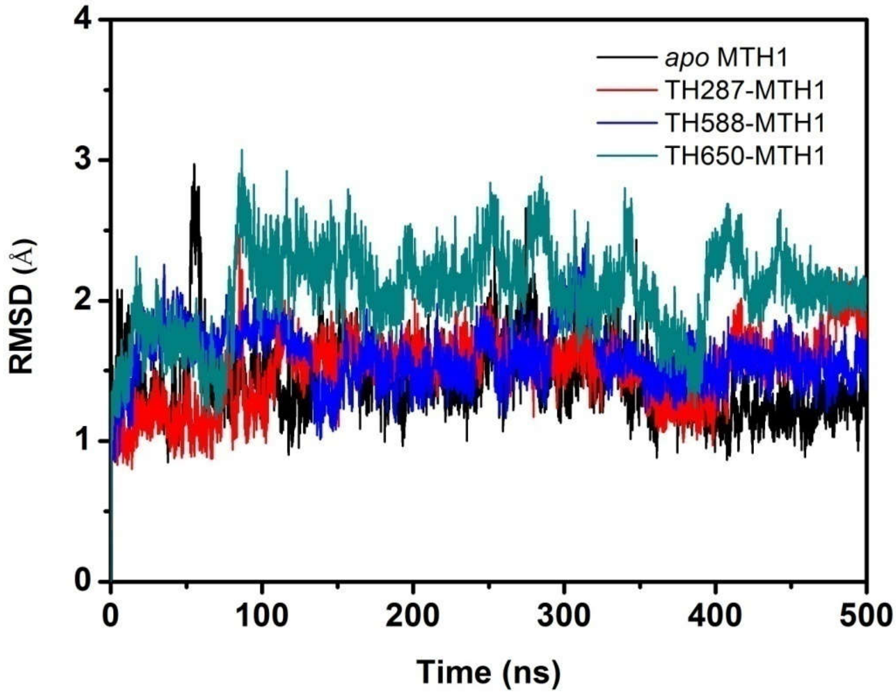

Figure S9. The Cα RMSD as a function of time for the *apo* MTH1, the TH287-MTH1 complex, the TH588-MTH1 complex and the TH650-MTH1 complex.
